# Membrane mediated phase separation of the bacterial nucleoid occlusion protein Noc

**DOI:** 10.1038/s41598-022-22680-5

**Published:** 2022-10-26

**Authors:** Leon Babl, Adrián Merino-Salomón, Nishu Kanwa, Petra Schwille

**Affiliations:** Max Planck Institute for Biochemistry, Am Klopferspitz 18, 82152 Planegg, Germany

**Keywords:** Biophysics, Biochemistry

## Abstract

Liquid–liquid phase separation is a fundamental biophysical process to organize eukaryotic and prokaryotic cytosols. While many biomolecular condensates are formed in the vicinity of, or even on lipid membranes, little is known about the interaction of protein condensates and lipid bilayers. In this study, we characterize the recently unknown phase behavior of the bacterial nucleoid occlusion protein Noc. We find that, similarly to other ParB-like proteins, CTP binding tightly regulates Noc’s propensity to phase separate. As CTP-binding and hydrolysis also allows Noc to bind and spread on membranes, we furthermore establish Noc condensates as model system to investigate how lipid membranes can influence protein condensation and vice versa. Last, we show that Noc condensates can recruit FtsZ to the membrane, while this does not happen in the non-phase separated state. These findings suggest a new model of Noc mediated nucleoid occlusion, with membrane-mediated liquid–liquid phase separation as underlying principle of complex formation and regulation thereof.

## Introduction

Compartmentalization of biochemical processes is a fundamental requirement to orchestrate the wealth of reactions necessary to maintain a functional cell. Long known strategies of compartmentalization involve the formation of membrane-encapsulated substructures within the cell’s interior. Prominent examples of such compartments are the eukaryotic nucleus or prokaryotic magnetosomes^1^. More recently, a novel strategy for cellular compartmentalization has gained significant attention: The formation of non-membrane bound organelles by protein or nucleic acid phase separation^2,3^.

Via a often enthalpically driven demixing of a homogenous solution into a component-dense and a component-dilute phase, biochemical reactions can be contained, accelerated and spatiotemporally controlled^4–6^. Highly complex cellular structures such as the nucleolus, a multilayered biomolecular condensate responsible for the production of functional rRNA, can be generated by phase separation of protein and nucleic acid components^7^. Recently, an increasing variety of cellular compartmentalization has been linked to liquid–liquid phase separation in eukaryotes^8^ and also prokaryotes^9^.

While our understanding of the assembly and control of these remarkable cellular features is advancing, fewer studies have focused on the interactions of membrane-bound and membraneless organelles^10–19^. However, many of the well-characterized phase separating systems are known to form in proximity and interact with lipid membranes in vivo^20,21^. For example, the prominent membraneless organelle of the germ line, the germ granule, forms and stays in vicinity to the nucleolar membrane for its entire lifespan^22^. Exciting theoretical studies have started to unravel principles of phase separation on membranes and other surfaces, predicting significant differences, such as a step-wise wetting transition, to bulk phase separation^23^. Interestingly, the interaction of synthetic aqueous-two phase systems, such as PEG-dextran mixtures, and membrane model systems have been described in depth and have shown to be able to deform, bud and organize biological membranes^24–26^. Also, binding of phase separating proteins to model membrane systems through artificial tags was shown to enhance membrane tubule formation, depending on the phase separated state of the bound proteins^15^. Synaptic proteins have been shown to co-phase separate with membrane vesicles, which has been interpreted as the mechanism of formation of the pre-synaptic density^13^. It is therefore becoming increasingly clear that many membraneless organelles interact with membrane bound compartments or the plasma membrane^20^.

So far, no bacterial condensates and lipid membrane interactions have been studied in vitro. However, recent studies have proposed that the bacterial ParB-protein family is capable of forming liquid–liquid phase separated condensates in various organisms^27–29^. While the largest part of the ParB-family are primarily DNA binding proteins, a subfamily is known to directly interact with lipid membranes. These so-called nucleoid occlusion proteins bind to the inner membrane leaflet and prevent the cell division machinery to form above the bacterial nucleoid^30^. Initially, a uniform distribution of Noc on the nucleoid was reported, but more recent studies revealed dynamic, foci-like structures of Noc on the membrane^31,32^. The formation of this higher-order complex and the mechanism of Z-ring inhibition above the nucleoid, however, are still poorly understood with little in vitro data to date.

Here we demonstrate that Noc, like other ParB proteins, undergoes a liquid–liquid phase separation depending on the environmental conditions. The phase separation is controlled by CTP-binding, and the formed droplets readily interact with and deform membranes. The formation of droplets, in reverse, is dependent on membrane characteristics, such as composition and order. Furthermore, phase separated Noc can up-concentrate FtsZ, potentially pointing to a physiological role of condensate formation in Z-ring regulation and highly similar to the *E. coli* nucleoid occlusion system^33^.

## Results

### Noc undergoes liquid–liquid phase separation

It was recently shown that a variety of ParB-like proteins undergo LLPS in vivo^27^ and in vitro^28^. Despite its different biological function, Noc’s ability to bind and spread on membranes is closely regulated by CTP-binding and hydrolysis^34^. This similarity to ParB lead us to hypothesize that Noc can also undergo CTP-controlled LLPS^28^. In accordance with this hypothesis, in vivo data suggests that the formed Noc nucleoprotein complex has a higher order structure and is highly dynamic^31,32^.

**Figure 1:**
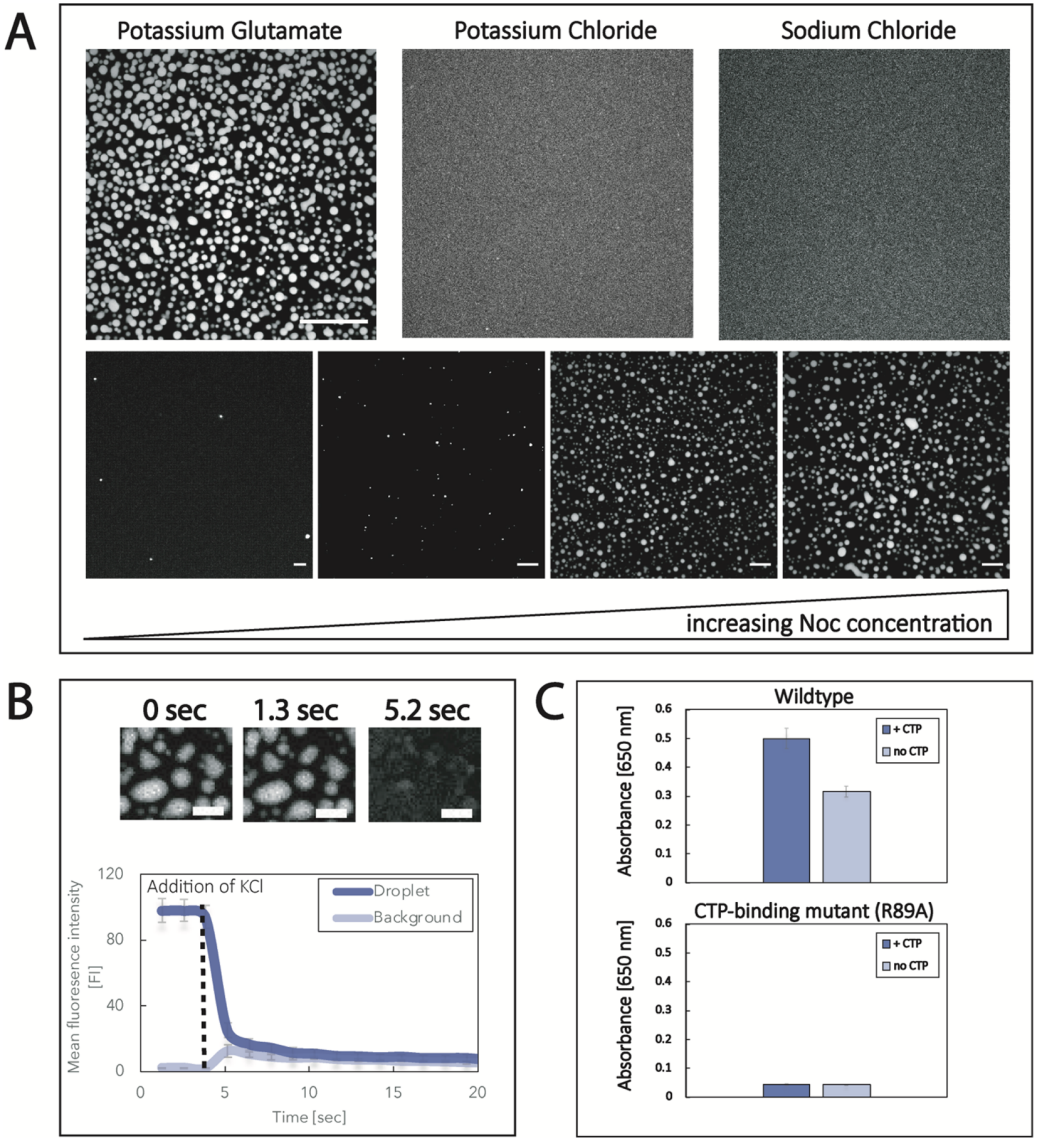
Noc undergoes liquid–liquid phase separation. (**A**) 8 µM of Atto488-labeled Noc in different buffers (20 mM Tris pH 7.4, 150 mM KGlu, KCl or NaCl and 5 mM MgCl_2_. The scalebar is 30 µm. Increase of concentration of Noc in KGlu-buffer leads to larger and more droplets. From left to right: 1 µM, 2 µM, 4 µM and 8 µM. The scalebar represents 20 µm. (**B**) Dissolution of droplets after addition of 500 mM KCl. The droplets were formed in KGlu-buffer and after formation, 500 mM KCl were added which resulted in rapid dissolution of the Noc droplets. Protein concentration was 8 µM and the scalebar represents 10 µm. Error bars are the standard deviation of three different droplet and background regions of interests. (**C**) Turbidity data: 20 µM of wildtype Noc was prepared in 20 mM Tris pH 7.4, 150 mM KGlu, 1 mM TCEP and 5 mM MgCl_2_ buffer and turbidity was measured with or without 1 mM CTP at 650 nm. Error bars represent the standard deviation of three replicates.

LLPS of proteins is greatly dependent on environmental conditions, such as ionic strength, buffer composition, or temperature^4,35^. Potassium glutamate is highly abundant in bacterial cells and has been shown to stabilize LLPS of bacterial proteins^28,36,37^. We therefore exposed fluorescently labeled Noc to a buffer containing 150 mM Potassium Glutamate and observed the formation of micrometer-sized spherical droplets (Fig. 1A). Replacing Potassium Glutamate with Potassium Chloride or Sodium Chloride does not lead to phase separation. Thus, Noc phase separation, similar to ParB^28^ and other bacterial proteins, is stabilized by Potassium Glutamate. The volume of phase separated droplets scales with protein concentration, as expected for LLPS (Fig. 1A). Noc droplets are highly dynamic, and the formation of the phase separated droplets is reversible as, upon addition of 500 mM KCl, the droplets rapidly dissolve (Fig. 1B). We also observed fusion of droplets and their relaxation into a spherical shape, indicating liquid-like behavior (Supp. Fig. 2 and Movie S1).

It was recently shown that ParB LLPS is tightly regulated by CTP-binding and that this regulation is conserved within the ParB protein family^28^. As Noc can also interact with CTP and its membrane binding is regulated by CTP-binding and hydrolysis, we ought to test whether CTP also stabilizes Noc LLPS^34^. Intriguingly, addition of CTP greatly increases the turbidity, indicating an increase in phase separated material. Also, a non-CTP-binding mutant (R89A) is unable to phase separate and shows no turbidity in absence and presence of CTP (Fig. 1C). These experiments demonstrate that CTP-binding also regulates Noc phase separation.

Taken together, we show that Noc undergoes LLPS depending on the environmental conditions, and the formation of droplets is regulated by CTP binding. These findings are remarkably similar to the properties of other ParB-protein droplets. However, the Noc subfamily has significant functional differences to most ParB-like proteins. Noc can bind to membranes to regulate the formation of the Z-ring. This is in stark contrast to the DNA-binding and nucleoid positioning functionality of many ParB-family members. We therefore sought to investigate if these biological differences manifest in the phase behavior of Noc.

### Noc condensates interact with membranes

While Noc and ParB share a common ancestry, their biological functionality greatly differs. This difference manifests in Noc’s ability to bind the bacterial chromosome to the plasma membrane to create nucleoid occlusion^38^. Recently, the role of CTP binding and hydrolysis in Noc membrane binding has been unraveled, and shown similar mechanisms to ParB binding and spreading on DNA^34,39^. These similarities lead us to conclude that Noc condensates could interact with biological membranes, making it a powerful model system to study the interaction of protein condensates with lipid membranes.

**Figure 2:**
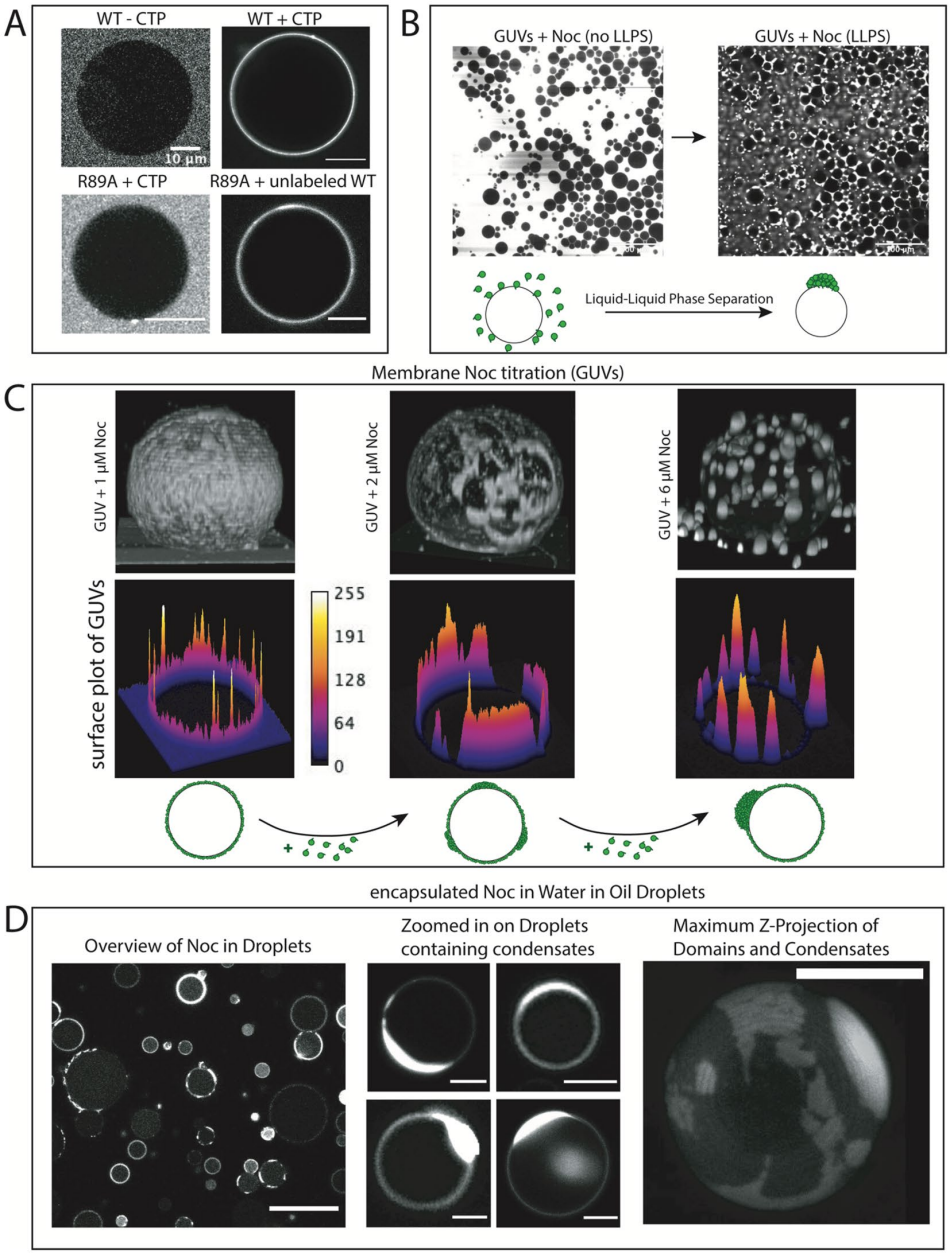
Noc condensates interact with membranes. (**A**) Noc and Noc mutants binding to GUVs (70 (POPC): 30 (POPG)). (1) 2 µM of Atto-488 Noc in absence of CTP and dsNBS does not bind to a GUV. (2) 500 nM of Atto-488 Noc binding to a GUV (70 (POPC): 30 (POPG)) in presence of 1 mM CTP and 1.5 µM dsNBS DNA in 20 mM Tris pH 7.4, 150 mM KGlu, 1 mM TCEP and 5 mM MgCl_2_ buffer. (3) Atto488-Noc^R89A^ mutant at 4 µM in presence of 1 mM CTP and 1.5 µM dsNBS does not bind to a GUV. (4) 3 µM of WT Noc recruit Atto488-Noc^R89A^ to the membrane. (**B**) Addition of 3 µM Atto-488 Noc to a sample containing GUVs (70 (POPC): 30 (POPG)). Buffer composition is 20 mM Tris pH 7.4, 150 mM KGlu, 1 mM TCEP, 1 mM CTP, 2.5 µM dsNBS and 5 mM MgCl_2_. The scale bar represents 100 µm. (**C**) Titration of Noc in samples containing GUVs (70 (POPC): 30 (POPG)) in presence of 1 mM CTP and 2.5 µM dsNBS. From left to right: 1 µM, 2 µM and 6 µM Atto488-Noc. Images are reconstituted using ImageJ 3D Viewer plugin. (**D**) Encapsulation of Noc in water-in-oil droplets. An apparent concentration 80 µM of Atto488-Noc was encapsulated in the water-in-oil droplets. However, the final concentration in the droplets is significantly lower, as Noc is partially lost during droplet encapsulation. Images on the left show an overview of several droplets. The middle image shows zoomed-in droplets with Noc condensates wetting the inside membrane. The image on the right shows a maximum Z-projection of an individual droplet containing a condensate and film-like structures. Scalebars represent 10 µm.

Initially, we verified the functionality of our fluorescence labeling strategy by binding Atto488-Noc to the outer membrane of giant unilamellar vesicles (GUVs), which serve as model systems to mimic the cell membrane. As recently shown, only in the presence of specific DNA sequence (dsNBS oligo) and CTP, Noc binds to the membrane^34^. The CTP-binding mutant, R89A, which is also deficient of LLPS, is unable to bind to the membrane in the presence of dsNBS and CTP. Interestingly, Atto488-Noc^R89A^ could be recruited to the membrane by addition of unlabeled wildtype Noc (Fig. 2A). This finding suggests that Noc can self-interact to recruit further molecules that are deficient of membrane binding, to the membrane. Similar self-interaction is a behavior often observed for phase separating proteins. To test if phase separated Noc interacts with membranes, we added 3 µM of Noc to a chamber containing GUVs (Fig. 2B). Upon phase separation, wetting, diffusion and fusion of condensates on GUVs was observed (Supp. Movie). This indicates that the formed condensates not only preferentially interact with membranes but remain liquid-like while binding them. To gain more insight into the formation of condensates on membranes, we performed a titration of Noc to GUVs. Interestingly, at lower concentrations of Noc, we observed the formation of film-like structures on the GUVs, whereas higher concentrations of Noc lead to the formation of round 3D-condensates (Fig. 2C). The condensate formation on the surface is specific to membranes, as we could not observe droplets or domain-like structures on Ni–NTA Agarose beads that also bound Noc on the surface (Supp. Fig. 5).

As Noc condensates are interacting with the inner, rather than the outer membrane in vivo^40^, we encapsulated Noc into water-in-oil droplets with a membrane monolayer. Interestingly, the encapsulation clearly leads to condensates wetting the inner membrane of the droplets and the formation of domain-like structures on the membrane, again demonstrating the preferential binding and phase separation of Noc on the membrane (Fig. 2D).

Taken together, these findings reveal that Noc condensates preferentially wet the surface of various membranes. Depending on the protein concentration, Noc can form film-like condensates or round 3D spheres on the membrane. When encapsulated into a membrane bound organelle, the condensates maximize the surface interaction with the membrane by wetting.

Biological membranes can vary immensely in composition, order and fluidity/flexibility^41^. We therefore sought to understand if membrane properties such as charge or flexibility alter Noc condensation. For this purpose, we introduced Noc to supported lipid bilayers (SLBs) as these allow more quantitative analysis of the formed droplets on the membrane.

**Figure 3:**
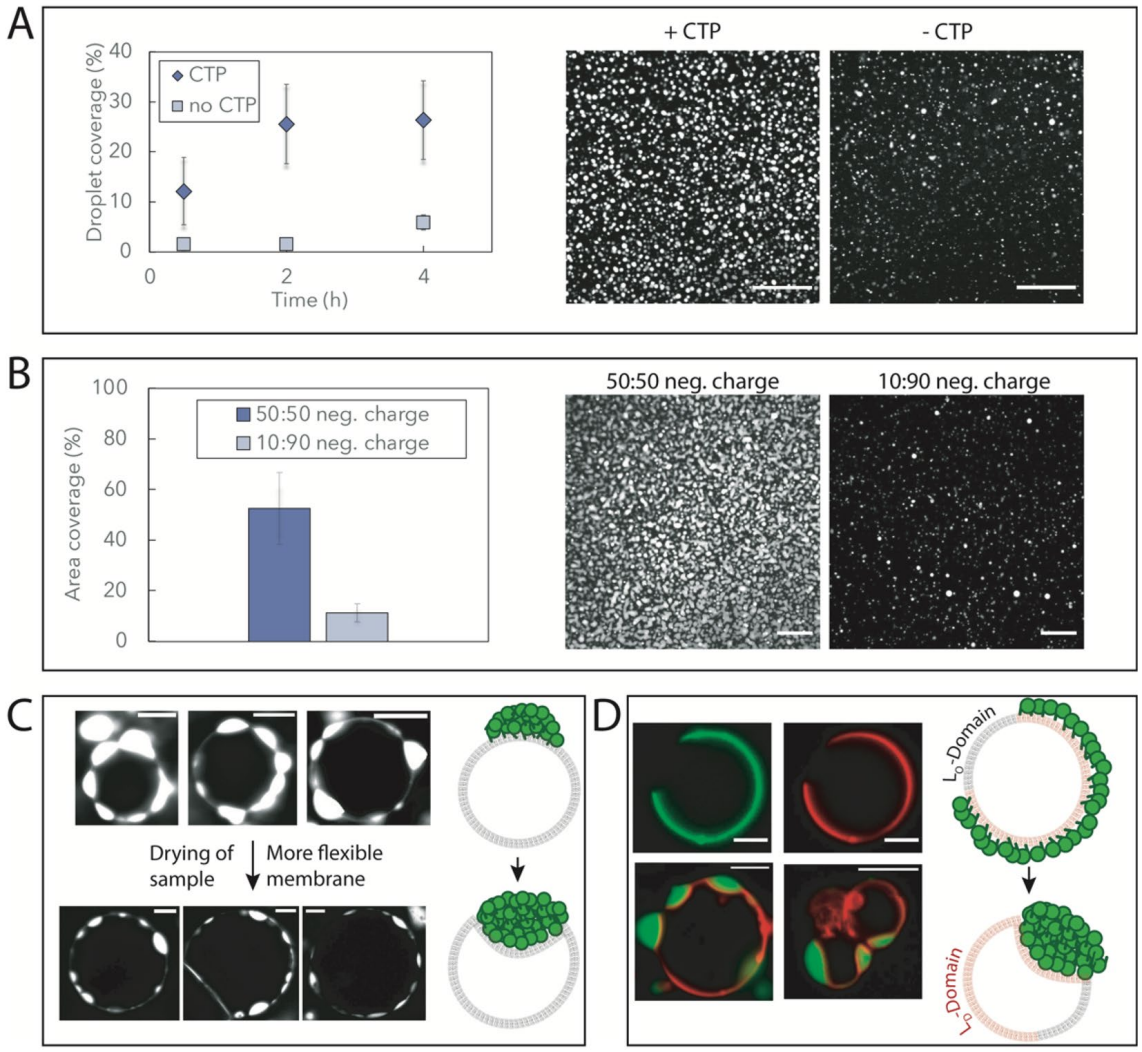
Noc condensates are influenced by membranes and can deform flexible membrane bilayers. (**A**) Droplet formation of Atto488-Noc on supported lipid bilayers (70:30 DOPC:DOPG) in presence and absence of CTP. The amount of phase separated material on the SLBs was monitored for 4 h. The area covered by droplets was calculated by thresholding the images and calculating the area fraction of the droplets. The images represent the amount of SLB coverage after 2 h and the scalebar is 50 µm. The protein concentration is 8 µM and the buffer is 20 mM Tris pH 7.4, 150 mM KGlu, 1 mM TCEP, 1 mM CTP, 2.5 µM dsNBS and 5 mM MgCl_2_. Error bars represent the standard deviation of three replicates. (**B**) Droplet formation of Atto488-Noc on supported lipid bilayers with different amounts of charged lipids (DOPG). The area covered by droplets was calculated by thresholding the images and calculating the area fraction of the droplets. The images represent the amount of SLB coverage after 2 h and the scalebar is 50 µm. The protein concentration is 16 µM and the buffer is 20 mM Tris pH 7.4, 150 mM KGlu, 1 mM TCEP, 1 mM CTP, 2.5 µM dsNBS and 5 mM MgCl_2_. Error bars represent the standard deviation of three replicates. (**C**) Noc condensates on GUVs (70:30 POPC:POPG) before and after drying the sample for 2 h. To allow evaporation of buffer, the oil layer above the buffer was removed. The protein concentration is 8 µM. The buffer is 20 mM Tris pH 7.4, 150 mM KGlu, 1 mM TCEP, 1 mM CTP, 2.5 µM dsNBS and 5 mM MgCl_2_. Scalebar 10 µm. (**D**) Noc condensates on phase-separated GUVs. The protein concentration is 2 µM (upper panel) and 12 µM (lower panel). The buffer is 20 mM Tris pH 7.4, 150 mM KGlu, 1 mM TCEP, 1 mM CTP, 2.5 µM dsNBS and 5 mM MgCl_2_. Scalebar 5 µm.

As Noc droplets also form in the absence of membrane binding (Fig. 1A), we compared a membrane bound to non-membrane bound state by adding CTP to the sample. We observed a significantly increased membrane coverage by phase separated droplets in the presence of CTP (Fig. 3A). While CTP generally stabilizes Noc phase separation, also in absence of a membrane, the measured difference between phase separated material in presence or absence CTP on SLBs is even higher than expected from the turbidity data (Fig. 1C) in bulk. It is therefore likely that membrane binding stabilizes the formation of Noc condensates.

Similarly, protein condensates are highly susceptible to changes in the direct electrostatic environment^35^. To test whether membranes can truly stabilize Noc LLPS, we prepared SLBs with different percentages of negatively charged lipids. Intriguingly, increasing the negative charge from 10 to 50% lead to a significant increase in phase separated material on the membrane (Fig. 3B). These findings provide evidence that membranes can alter the formation of condensates by affecting the immediate electrostatic environment.

This opens the question if vice versa, protein condensates can influence lipid membranes. For example, it has been demonstrated that protein condensates can cause GUV tubulation when artificially bound to the membrane, and synthetic aqueous two phase systems have been shown to cause membrane bending^15,42^. We therefore slowly dried GUV containing samples to cause an osmolar mismatch with the surrounding buffer. This mismatch creates more flexible membranes as water leaves the GUVs, lowering the bending rigidity of the membrane and making it more flaccid in nature. While Noc condensates on non-flexible membranes are clearly pointing outwards, upon drying the membrane becomes flexible and the condensates point into the GUVs (Fig. 3C).

To mimic more complex membrane compositions often found in vivo^41,43^, we performed similar experiments with membrane-phase separated GUVs to account for the presence of membrane rafts in the cells. Based on the lipid composition, GUVs partition into liquid-ordered (L_o_) and liquid-disordered (L_D_) domains. Interestingly, Noc was found to colocalize with the L_D_-domains (red-colored), which are known to be more flexible. Upon increasing Noc concentration, Noc condensates were also only observed on the L_D_-domains of the GUVs and clearly deformed the phase separated GUVs at high protein concentrations (Fig. 3D). This preferential binding to L_D_ domains potentially results from the higher fluidity of L_D_ domains as compared to the more rigid L_o_ domains, accumulating more protein and facilitating the phase transition at the L_D_ domains.

Thus, Noc condensation on membranes is influenced by the protein affinity towards the membrane, as well as the membrane’s physical properties. Together with the film-like structures (Fig. 2) depending on Noc concentration, these findings point towards a surface mediated condensation for the assembly of Noc droplets on membranes. Once formed, Noc condensates can deform membranes similar to recent findings of tubule formation by protein phase separation on GUVs^15^.

### Noc condensates can recruit FtsZ to lipid membranes

Last, we investigate the potential role of Noc condensates in *B. subtilis* nucleoid occlusion. The *E. coli* nucleoid occlusion factor SlmA has been shown to form liquid-like condensates which absorb FtsZ and hinder its polymerization^33^. The conservation of liquid condensate formation between SlmA and Noc is particularly intriguing, as the two proteins are not known to share similarities in mechanism and protein structure. While SlmA has been demonstrated to bind to FtsZ and alter its polymerization dynamics, Noc is thought to not or very weakly interact with FtsZ.

**Figure 4:**
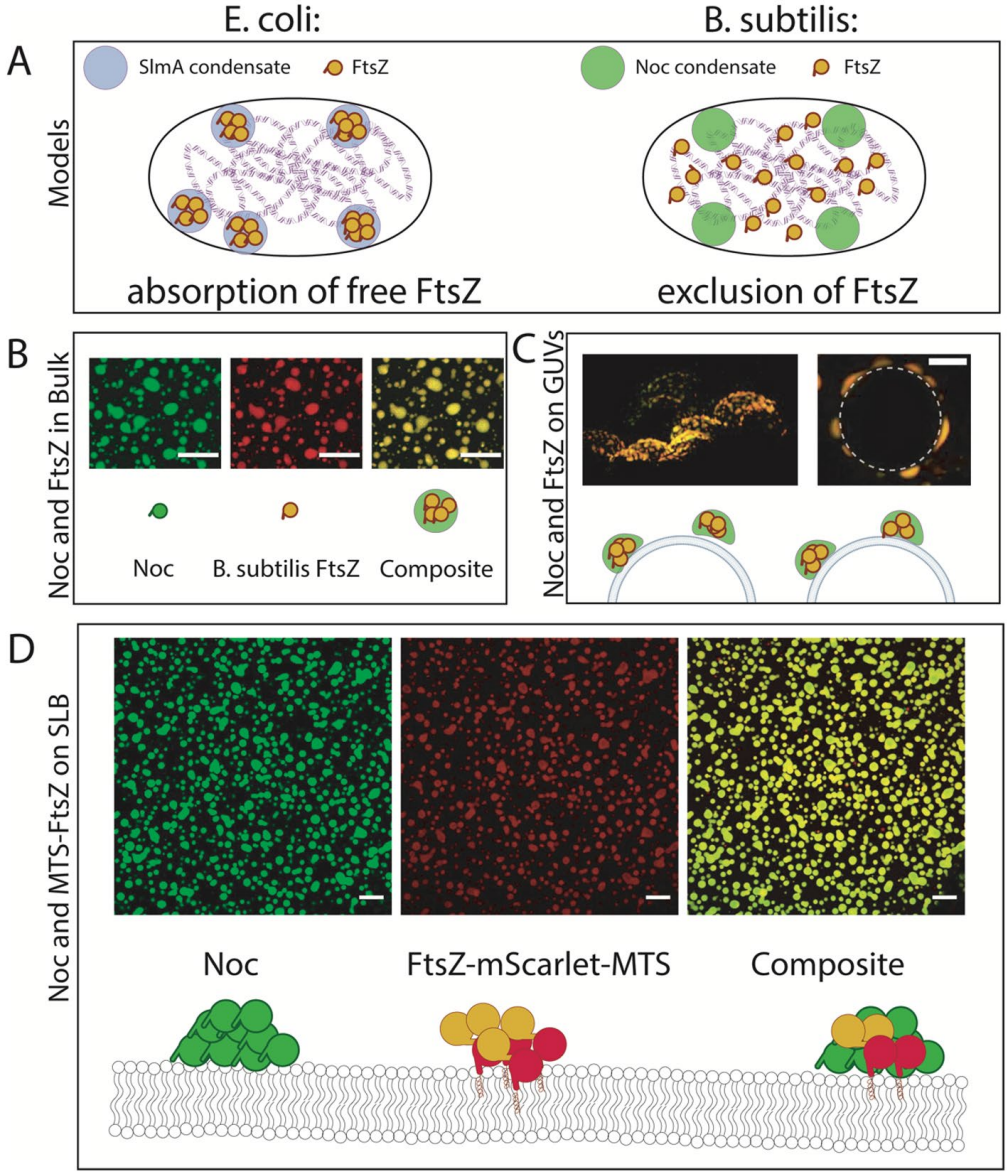
Noc condensates non-specifically up-concentrate FtsZ on the membrane and in bulk. (**A**) Current models of Nucleoid occlusion in E. coli (left) and B. subtilis (right). (**B**) Partitioning of B. subtilis FtsZ into Noc droplets in bulk. Left: 8 µM Noc, 10 µM B. subtilis FtsZ^Alexa647^ Buffer conditions: 50 mM Mes pH 6.5, 50 mM KCl, 10 mM MgCl_2_, 1 mM EDTA and 2 mM GTP. The scalebar represents 10 µm. (**C**) Partitioning of B. subtilis FtsZ into Noc droplets on GUVs. 4 µM Noc, 1 µM FtsZ in 20 mM Tris pH 7.4, 150 mM KGlu, 5 mM MgCl_2,_1 mM CTP and 2.5 µM dsNBS. The GUVs consist of 70:30 POPC:POPG. Scalebar 10 µm. (**D**) Partitioning of B. subtilis FtsZ-mScarlet-MTS on SLBs. Noc concentration is 5 µM and 600 nM FtsZ-mScarlet-MTS. Buffer conditions: 50 mM Mes pH 6.5, 50 mM KCl, 10 mM MgCl_2_, 1 mM EDTA, 5 mM GTP, 1 mM CTP and 2.5 µM dsNBS. The SLB consists of 70:30 DOPC:DOPG lipids. Scalebar 50 µm.

In accordance with previous findings^40^, we could not observe a relevant direct binding using labeled FtsZ and titrating Noc in microscale thermophoresis experiments. Using quartz crystal microbalance (QCM) experiments, we quantitatively compared membrane coverage in presence and absence of FtsZ and could not detect significant additional protein attachment in presence of *B. subtilis* FtsZ and Noc, compared to a control only containing Noc (Supp. Fig. 3). Also, FtsZ GTPase activity is not altered by titrating in Noc, in presence and absence of dsNBS DNA (Supp. Fig. 4). These findings demonstrate that Noc and FtsZ do most likely not or only weakly interact, and Noc does not recruit FtsZ to lipid bilayers in the non-phase separated state. However, it is known that biomolecular condensates can utilize weak (high µM to low mM K_D_) affinities to significantly up-concentrate molecules^44^. We therefore tested if Noc condensates can concentrate FtsZ, similar to the *E. coli* orthologue SlmA. Upon phase separation of Noc, strong partitioning of labeled FtsZ was observed (Fig. 4B). Similarly, non-membrane bound FtsZ was strongly partitioned into Noc droplets on GUVs (Fig. 4C). This is most likely a result of the high local Noc concentration, offering a plethora of interactions to FtsZ and therefore potentiating any weak interaction. In vivo, however, FtsZ is bound to the membrane by FtsA and SepF^45–47^. We therefore constructed a membrane-bound version of FtsZ (FtsZ-mScarlet-MTS) and measured its partitioning into Noc droplets on SLBs. Expectedly, the membrane-bound FtsZ, FtsZ-mScarlet-MTS, was also strongly up-concentrated within the membrane-bound Noc droplets (Fig. 4D). Interestingly, Noc droplets can also up-concentrate *E. coli* FtsZ, suggesting that the Noc-FtsZ interaction is located at the conserved regions of FtsZ (Supp. Fig. 6).

To summarize, we show that Noc in a non-phase separated state does not recruit or enrich FtsZ on the membrane, however upon phase separation it recruits FtsZ to the membrane. These findings are potentially pointing towards a regulative role of Noc LLPS in nucleoid occlusion, intriguingly in high similarity to the *E. coli* system^33^.

## Discussion

Self-organization of ParB-like proteins is an increasingly studied topic. A variety of members from this protein family have been shown to undergo liquid–liquid phase separation in vitro and in vivo^27,28,48^. We demonstrate that a functionally unrelated ParB-like protein, Noc, also undergoes LLPS. Intriguingly, the mechanism of regulation of phase separation appears to be conserved among the ParB-family members. Noc condensates physically interact with biological membranes, resulting in condensate and membrane deformations. Likewise, membrane composition and order can influence condensation. Lastly, we show that Noc condensates up-concentrate FtsZ in bulk and on membranes, pointing towards a regulatory mechanism of Noc condensation in Z-ring assembly.

The formation of liquid-like condensates is supposed to be a crucial mechanism in cellular compartmentalization^8^. While we know that protein evolution can impact on sequence, structure and function, it is not yet clear if similar mechanisms are in place for the phase properties of proteins. In eukaryotes, it has been hypothesized that an individual protein family, the DEAD-box helicases, regulates a large fraction of the biomolecular condensates found in vivo^49^. However, this protein family only has few prokaryotic members. We therefore recently suggested that the prokaryotic ParB protein family could serve a similar role in bacteria, as several of these proteins, or proteins with similar functionality, have been shown to undergo liquid–liquid phase separation^28,50,51^. To scrutinize this hypothesis, we purified the functionally unrelated ParB-like protein, Noc, and showed that this protein also undergoes LLPS. Intriguingly, Noc phase separation is also regulated by CTP, as previously shown for ParB. These similarities of phase properties and regulation thereof are an excellent example of the potential evolutionary conservation of LLPS within protein families.

However, a striking difference between Noc and ParB is Noc’s N-terminal membrane binding helix. Through regulation by CTP hydrolysis and DNA binding, Noc can bind and spread on membranes^34^. This mechanism of binding and spreading is highly similar to ParB binding and spreading on DNA. These similarities led us to the conclusion that Noc condensates could interact with, and potentially be regulated by, lipid membranes. While recent advances related more biomolecular condensates with membrane binding, only few studies systematically investigate the influence of membranes on condensates, and vice versa^12,15,20^. Intriguingly, we found that membrane binding, membrane composition and membrane order can regulate the formation of condensates. These findings point towards a coupling of membrane- and protein phase separation, which likely plays a fundamental role in the organization of biological condensates in vivo. Also, forces generated by protein condensates acting on membranes have attracted attention and could be of broad interest, for example as recently shown for endocytosis^52,53^. The formation of film- or domain-like structures on GUVs and in water-in-oil droplets by Noc is highly similar to recent reports of phase separating proteins on membrane surfaces, where domain-like architectures were observed^15,17^. These findings indicate a wetting-like transition of Noc on the membrane, as recently shown for a DNA-binding protein^54^ and studied theoretically^23^.

Taken together, we demonstrate that Noc readily forms condensates on a variety of model membranes, governed by liquid–liquid phase separation. We show that membrane properties such as charge or fluidity alter the condensate formation. In accordance with recent reports of artificial coacervates bending and wetting membranes, we observe that Noc condensates generate forces on membranes, deforming and wetting a variety of model membranes. Therefore, Noc droplets represent a new and exciting class of biomolecular condensates, which not only interact with membrane-bound compartments but are also “out-of-equilibrium” through constant CTP-hydrolysis. We hope that the described findings will catalyze novel theoretical and experimental studies, advancing our understanding of out-of-equilibrium protein condensation on biological surfaces.

## Methods and materials

### Protein purification

#### Noc and Noc mutants

Noc and its R89A mutant were purified as previously described^34^. In brief, protein was expressed in *E. coli* Rosetta (DE3) cells in LB medium at 37 °C supplemented with carbenicillin. Protein expression was induced at OD of ~ 0.6 after cooling the culture to 4 °C with 1 mM IPTG. Expression was continued for 3–4 h at 28 °C. Cells were spun down and lysed by sonication. The cell debris was removed through centrifugation at 28,000 g for 30 min at 4 °C. The lysate was then loaded into a 5-mL HisTrap column (GE Healthcare) that had been equilibrated with buffer A [100 mM Tris–HCl pH 8.0, 250 mM NaCl, 10 mM imidazole, and 5% (v/v) glycerol]. Protein was eluted from the column using an increasing gradient of imidazole (10–500 mM) in the same buffer. Noc-containing fractions were pooled together and diluted to a conductivity of 16 mS/cm before being loaded onto a 5-mL Heparin HP column (GE Healthcare) that had been equilibrated with 100 mM Tris–HCl pH 8.0, 25 mM NaCl, and 5% (v/v) glycerol. Protein was eluted from the Heparin column using an increasing gradient of NaCl (25 mM to 1 M) in the same buffer. Noc was further purified using a Superdex-75 gel filtration column (GE Healthcare). The gel filtration column was pre-equilibrated with buffer containing 10 mM Tris–HCl pH 8.0, 250 mM NaCl and 5 mM MgCl_2_. Eluted protein fractions were analyzed for purity by SDS-PAGE. Mutants were purified with the same protocol, for the KCK-tag variant for fluorescent labeling, TCEP (1 mM) was added prior to labeling and for storage.

#### *B. subtilis* FtsZ

The protein was purified as described elsewhere^55,56^. In brief, the protein was expressed in *E. coli* C41 (DE3) cells at 37 °C in LB medium to an OD_600_ of 0.6. Expression of the protein was induced with 1 mM IPTG and the cells grown for another 4 h at 37 °C. Cells were harvested by centrifugation and washed with 50 mM Tris pH 8.8, 100 mM NaCl, 1 mM EDTA. Cells were lysed by three rounds of sonication in 50 mM Tris pH 8.8, 100 mM NaCl, 1 mM EDTA and lysate was cleared by spinning at 160,000xg for 45 min at 4 °C. All subsequent steps were carried out at 4 °C. Protein was precipitated by slowly adding 0.43 equivalents of saturated Ammonium sulfate solution and incubation on ice for 20 min. Precipitates were separated by centrifugation at 10.000 g at 4 °C for 10 min. The supernatant was kept, and another 0.16 equivalents of Ammonium sulfate were slowly added. After another 20 min incubation on ice, the protein was spun down. The pellet was resuspended in 50 ml of 50 mM Tris pH 8.5, 50 mM KCl, 1 mM EDTA, 1% sucrose and loaded onto a GE HealthCare MonoQ 10/100 column. The protein was eluted by applying a gradient with 50 mM Tris pH 8.5, 500 mM KCl, 1 mM EDTA, 1% sucrose to the column. Protein was then dialyzed into 50 mM HEPES pH 7.5, 50 mM KCl, 2.5 mM MgCl_2_, 1 mM EGTA and 10% sucrose, frozen in liquid nitrogen and stored at − 80 °C.

#### *E. coli* FtsZ

*E. coli* FtsZ purification: FtsZ was purified by following the calcium precipitation method previously described^57^. Briefly, FtsZ was expressed in C21 cells in LB medium at 37 °C. Protein expression was induced with IPTG for 3 h followed by centrifugation and lysis of the cells by sonication. Cell debris was removed by centrifugation at 50.000 g using an MLA 80 rotor at 4 °C for 1 h. FtsZ was polymerized by adding 1 mM GTP and 20 mM CaCl_2_ to a buffer containing 50 mM PIPES 5 mM MgCl_2_, 1 mM EDTA_KOH_ pH 6.5 followed by incubation for 15 min at 30 °C using a water bath. FtsZ Polymers were spun down at 16.000 g in ML-80 for 15 min at 4 °C and subsequently resuspended in buffer without GTP and CaCl_2_, disassembling the polymers into the FtsZ monomeric state. 1 mM GTP and 20 mM CaCl_2_ was added again to promote FtsZ polymerization a second time. FtsZ was incubated at 30 °C for 15 min and FtsZ polymers were spun down and resuspended afterwards as described above. Resuspended FtsZ was centrifuged at 16.000 g for 15 min at 4 °C. The remaining supernatant was loaded onto a Hi-TRAP Q-Sepharose column and the protein was eluted from the column by using a gradient of KCl (25 mM–1 M). Fractions of FtsZ were pooled and frozen at − 80 °C. FtsZ concentration and purity were measured by absorbance at 280 nm and SDS-PAGE respectively.

### Protein labeling

#### Noc Atto488 maleimide:

Atto488 maleimide was bought from SigmaAldrich (Cat. Nr. 28,562) KCK-tagged Noc was purified as described and cysteines reduced by addition of 1 mM TCEP. Labeling was achieved by following the manufacturer’s instruction. Labeling efficiency was determined to be ~ 20%.

#### *B. subtilis* Alexa-Fluor 647 NHS Ester:

Protein was dialyzed into 20 mM HEPES pH 8.0, 1 mM EDTA, 50 mM KCl and 5 mM MgCl_2_. Protein concentration was adjusted to 250 µM and 350 µg Alexa-Fluor 647 NHS-Ester dissolved in DMSO were added. The sample was incubated at room temperature (22 °C) for 45 min. Free dye was separated by a gravity flow column (equilibrated in 50 mM Tris pH 7.9 50 mM KCl 10% Glycerol, 2.5 mM MgCl_2_ and 1 mM EGTA). Fractions containing FtsZ were pooled and flash frozen in liquid nitrogen.

#### *E. coli* NHS-647

E. coli FtsZ was covalently labelled in the amine groups with Alexa 488 NHS ester as previously described^58,59^. FtsZ was labeled at its polymerized state by mixing FtsZ in presence of 1 mM GTP and 10 mM of CaCl_2_ with a 1:4 molar excess of Alexa 488 dye for 15 min at 30 °C followed by ultracentrifugation for 15 min at 19,000 g to remove inactive protein. Labelled FtsZ polymers were resuspended in cold buffer to favor depolymerization and incubated for 20 min at 4 °C. Free dye was separated by a HiTrap Desalting column (GE Healthcare). The degree of labeling of different fractions was estimated by absorbance and the protein was flash frozen at − 80 °C in 50 mM Tris–HCl, 150 mM KCl, 5 mM MgCl_2_, and 10% Glycerol pH 7.5.

### Molecular cloning

All constructs were designed and cloned using a seamless cloning strategy. In brief, vectors were linearized using indicated primers (Table S1) in a PCR (Phusion Polymerase (ThermoFisher Scientific Cat. No. F530L)). Following PCR, template was digested using DpnI (NEB) at 37 °C for 30 min. PCR products were visualized by agarose gel electrophoresis (agarose 0,8%, TAE, 120 V, 40 min), cut-out and purified using a QIAGEN gel extraction kit (Cat. No. 28704). Inserts were amplified by PCR using the appropriate primers (Table S1). Vector and insert were assembled by seamless cloning using the ThermoFisher Scientific /Invitrogen GeneArt™ Seamless Cloning and Assembly Enzyme Mix (A14606) and transformed into chemically competent OneShot Top10-cells (Cat. No. C404006). Selection for successful cloning was done on LB-Ampicillin or LB-Kanamycin plates. Cells were grown overnight at 37 °C. Then, individual clones were picked and grown overnight in LB media (5 ml) containing the appropriate antibiotic (Ampicillin or Kanamycin). Plasmids were purified by a Miniprep kit (Cat. No.27104) and sequenced to verify successful cloning.

### GUV preparation

#### Double emulsion (homogeneous GUVs)

Lipid vesicles were produced by the double emulsion method^60^ with slight modifications. Briefly, 1-palmitoyl-2-oleoyl-glycero-3-phosphocholine (POPC) and 1-palmitoyl-2-oleoyl-sn-glycero-3-phospho-(1'-rac-glycerol) (POPG) (Avanti Polar Lipids, Alabaster, AL, USA) were mixed and dissolved in chloroform at different molecular ratios (70:30) with a final concentration of 25 g/L. For fluorescence visualization of the vesicle membrane, 0.005% of 1,2-dioleoyl-sn-glycero-3-phosphoethanolamine (DOPE) labelled with ATTO 655 (ATTO-Tech GmbH, Siegen, Germany) was included to the lipid mixture. 100 μL of the lipid mixture was dried under N_2_ and re-dissolved in 25 μL of decane (TCI Deutschland GmbH, Eschborn, Germany). 1 mL of mineral oil (Carl Roth GmbH, Karlsruhe, Germany) was supplemented to the mixture and thoroughly vortexed for ~ 1 min. 50 μL of this lipid-in-oil mixture was carefully added on top of 100 μL of reaction buffer in a 96-Well Flat-Bottom Microplate (SensoPlate, Greiner Bio-One GmbH, Kremsmuenster, Austria) previously passivated with 5 g/l of casein for ~ 30 min. This mixture was incubated for ~ 20–30 min to favor the formation of a lipidic monolayer. At the same time, the inner solution containing 30 mg/mL Ficoll and 10 mg/mL BSA suspended in reaction buffer was prepared. Subsequently, 2.5 μL of inner solution was added to a 100 μL of lipid-in-oil in a 1.5 mL Eppendorf tube and water-in-oil emulsion was formed by tipping the tube thoroughly. 80 μL of this emulsion was then carefully dripped on top of the previously formed monolayer and subsequently centrifuged for 10 min at 1000 g to obtain lipid vesicles. After centrifugation, the 96 well-plate allowed the direct visualization of lipid vesicles on the confocal microscope.

#### Electroformation (phase-separated GUVs)

Phase separated Giant Unilamellar Vesicles (GUVs) utilized throughout this work were prepared by electroformation in PTFE chambers with Pt electrodes, as previously described elsewhere with minor modifications^61^. The lipid composition chosen was DOPC: DOPG: DSPC: Cholesterol (40:20:20:20) doped with 0.1 mol% Atto655-DOPE to introduce phase separation and negative charge. Briefly, 6 μL of the lipid mixture (2 mg mL^−1^ in chloroform) was spread onto two Pt wires to make a thin film and dried in a desiccator for 30 min. The PTFE chamber was filled with 350 μL of an aqueous solution of sucrose with approximate 340 mOsm kg^−1^ osmolarity (iso-osmolar compared to the imaging buffer). While keeping the PTFE chambers at 60 °C, an AC electric field of 2 V (RMS) was applied at a frequency of 10 Hz for 1 h, followed by 2 Hz for 0.5 h. The chambers were allowed to cool down to room temperature before performing any experiments.

### Water-in-oil droplets

A mixture of lipids for a final volume of 500 μl (70:30 DOPC:DOPG; 0.01% ATTO655-DOPE) was prepared at a concentration of 1.5 mg/ml and dried under nitrogen. 10 μl of Decane was added and the solution was vortexed until a turbid solution was achieved. 500 μl of mineral oil was added and aliquoted in 5 × 100 μl. 1 μl of inner solution containing 20 mM Tris pH 7.4 150 mM KGlu, 5 mM MgCl_2_, 1 mM CTP, 1 mM TCEP and 1.5 μM dsNBS plus the appropriate Noc-Atto488 concentration was added. Droplet formation was achieved by thoroughly vortexing the sample. Imaging was done in 96-Well Flat-Bottom Microplate (SensoPlate, Greiner Bio-One GmbH, Kremsmuenster, Austria) previously passivated with 5 g/l of casein for ~ 30 min.

### Supported lipid bilayer (SLB) preparation

SLBs were formed via vesicle fusion. Coverslips were rinsed with ethanol and distilled water, and surface-etched with oxygen plasma (30 s at 0.3 mbar, Zepto, Diener Electronics). Lipids dissolved in chloroform were mixed in glass vials, and after evaporation of the solvent under a gentle N_2_ stream, the lipids were re-suspended in SLB formation buffer (25 mM tris, 150 mM KCl, 5 mM MgCl_2_, pH 7.5) to 4 µg/µl, and vortex until the lipid films are completely resuspended, forming a cloudy solution containing Multilamellar Vesicles (MLVs) of various sizes. The obtained large multilamellar vesicle suspensions were then sonicated until solutions were clear. These small unilamellar vesicle solutions were either stored at − 20 °C and re-sonicated before use or used immediately. The sonicated small unilamellar vesicle solutions diluted to ca. 0.5 µg/µl in SLB formation buffer were added into liquid chambers pre-warmed to 37 °C, filling the chamber. After 5 min incubation at 37 °C, liquid chambers now containing SLBs were washed with 10 × 200 µl SLB washing buffer (25 mM Tris pH 7.5, 150 mM KCl).

### QCM-D

Prior to each measurement, silicon dioxide (SiO_2_)-coated quartz crystal sensors (Biolin Scientific, Gothenburg, Sweden) were treated with a 3:1 mixture of sulfuric acid and hydrogen peroxide (piranha-solution). Subsequently, sensors were rinsed with ultrapure water, dried under a stream of nitrogen, and mounted in the flow modules of the Qsense Analyzer (Biolin Scientific, Gothenburg, Sweden). After baseline stabilization, supported lipid bilayer formation (SLB) was induced through constant injection (flow rate: 0.15 mL/min) of a 1 mg/mL mixture of small unilamellar vesicles (DOPC/DOPC, 70:30 mol %) in buffer (25 mM Tris- HCl pH 7.5, 150 mM KCl, 5 mM MgCl_2_), spiked with 5 mM CaCl_2_. The formed bilayer was washed with buffer until no frequency change was observed. Then, 150 µl of sample was flown over the sensor at 0.1 ml/min and the change in frequency monitored at overtone F9.

### Turbidity

80 µl of sample were prepared by mixing protein with the buffer containing crowding agents and molecules of interest in 384 Greiner Black Flat Plates. After 10 min of incubation, turbidity was measured using a TECAN plate reader at room temperature at 650 nm.

### Microscopy

All images were taken on a Zeiss LSM780 confocal laser scanning microscope using a Zeiss C-Apochromat × 40/1.20 water-immersion objective (Carl Zeiss). Longer time series were acquired using the built-in autofocus system. Noc Atto 488 was excited using a 488 nm argon laser and FtsZ-Alexa 647 using a 633 nm He–Ne laser. Images were typically recorded with a pinhole size of 2.6–4 Airy units for the channels 512 × 512-pixel resolution and a pixel dwell time of 1.27 μs. Line-averaging was set to 2. Images were analysed in ImageJ^62^.

## Supplementary Information

Supplementary Video 1.

Supplementary Video 2.

Supplementary Video 3.

Supplementary Information 1.

Supplementary Information 2.

## Abbreviations

GUV: Giant unilamellar vesicle
SLB: Supported lipid bilayer
LLPS: Liquid–liquid phase separation
QCM: Quartz crystal microbalance
