# Supplementary material for: Membrane mediated phase separation of the bacterial nucleoid occlusion protein Noc
Source: Sci Rep. 2022 Oct 26;12:17949. doi: 10.1038/s41598-022-22680-5 (PMC9606368; doi:10.1038/s41598-022-22680-5)
Supplement: Supplementary file 4 — Supplementary Information 1. [file 41598_2022_22680_MOESM4_ESM.docx]

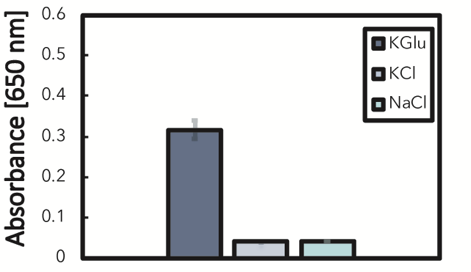


Suppl. Figure 1: Turbidity of Noc in different salts. 20 µM wildtype Noc was introduced into 20 mM Tris pH 7.4 with either 150 mM KGlu, KCl or NaCl with 5 mM MgCl_2_ and turbidity was measured at 650 nm. Error bars represent the standard deviation of three replicates.


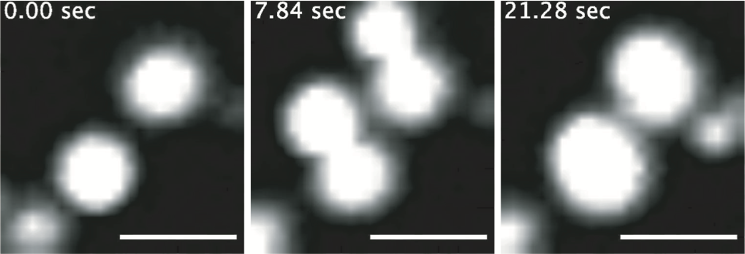


Suppl. Figure 2: Fusion of Noc droplets. The scalebar represents 5 µm.


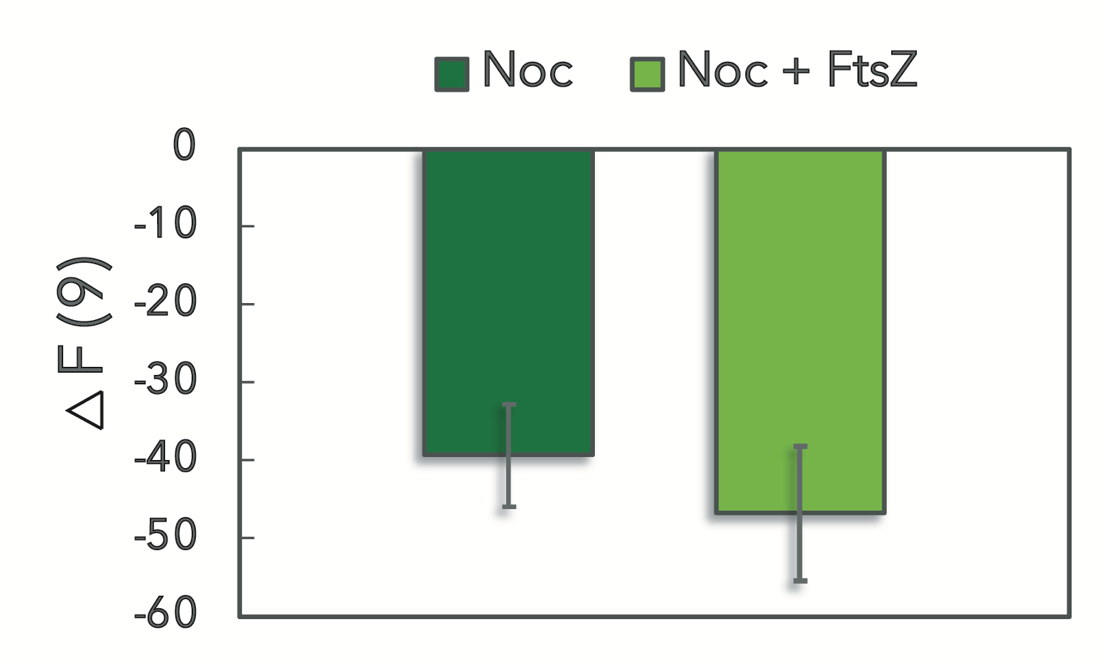


Suppl. Figure 3: Comparison of QCM frequency shift between Noc and Noc + FtsZ binding to a supported lipid bilayer. Protein concentrations were 5 µM Noc and 6 µM FtsZ. The buffer contained 50 mM Tris pH 7.4, 150 mM KCl, 5 mM MgCl_2_, 5 µM dsNBS, 1 mM CTP and 1 mM GTP. Error bars represent the standard deviation of three replicates. The difference between Noc and Noc + FtsZ is not statistically significant (two-tailed t-test).
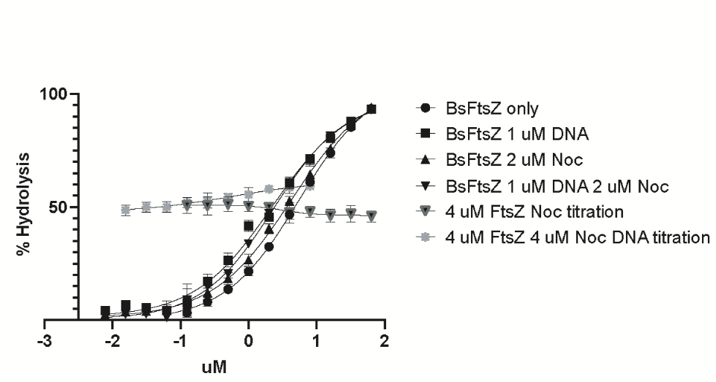


Suppl. Figure 4: GTPase activity of B. subtilis FtsZ in presence of Noc, dsNBS and Noc +dsNBS. No significant difference in hydrolysis rate is observed for all tested scenarios. Either Noc was titrated into the solution and FtsZ concentration was kept constant, or FtsZ was titrated, and Noc/DNA concentration was maintained constant.


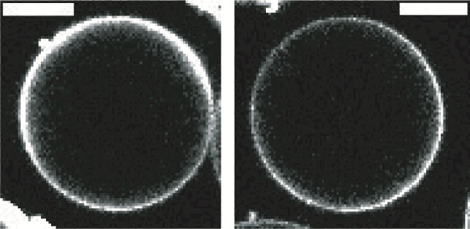


Suppl. Figure 5: Ni-NTA Agarose beads with Noc (3 µM) bound to the surface do not show condensates on the beads.


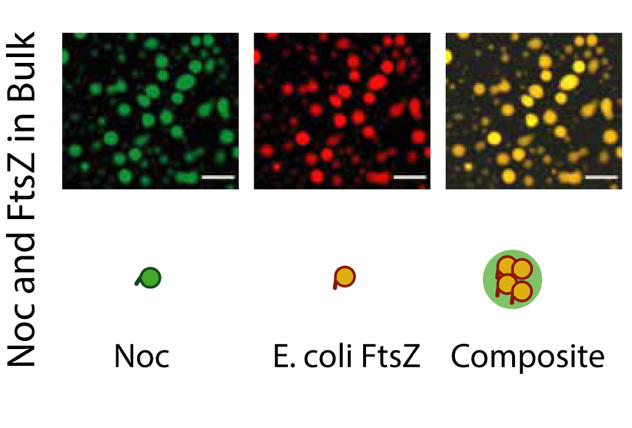


Suppl. Figure 6: Partitioning of E. coli FtsZ into Noc droplets in bulk 8 µM Noc, 5 µM E. coli FtsZAlexa647 20 mM Tris pH 7.4, 150 mM KGlu, 5 mM MgCl2 and 2 mM GTP. Scalebar 10 µm.
