# Supplementary material for: Membrane mediated phase separation of the bacterial nucleoid occlusion protein Noc
Source: Sci Rep. 2022 Oct 26;12:17949. doi: 10.1038/s41598-022-22680-5 (PMC9606368; doi:10.1038/s41598-022-22680-5)
Supplement: Supplementary file 5 — Supplementary Information 2. [file 41598_2022_22680_MOESM5_ESM.docx]

| Construct | Vector fwd | Vector rev | Insert fwd | Insert rev |
| --- | --- | --- | --- | --- |
| pET21a_KCK-Noc | Template: pET21a_Noc wt  ATAAAGTTGCAGGACCACTTCTG | Template: pET21a_Noc wt  CAGAAGTGGTCCTGCAACTTTAT | Template: pET21a_Noc wt  aaatgcaagCACCACCACCACCACCAC | Template: pET21a_Noc wt  cttgcatttCTCGAGTGCGGCCGCAAGCT |
| pET21b_Noc- R89A-KCK-His | Template: pET21b_Noc R89A  ATAAAGTTGCAGGACCACTTCTG | Template: pET21b_Noc R89A  CAGAAGTGGTCCTGCAACTTTAT | Template: pET21b_Noc R89A  aaatgcaagCACCACCACCACCACCAC | Template: pET21b_Noc R89A  cttgcatttCTCGAGTGCGGCCGCAAGCT |
| pET11b_FtsZbsu-mScarlet-I-MTS | Template: pET11b_FtsZbsu-Venus-MTS  gcggccgcctgcaggtcg | Template: pET11b_FtsZbsu-Venus-MTS  ggcggccgcttcattgaagaagag | Template: pCoofy_mScarlet-I  aggcggccgcATGGTTTCTAAAGGTGAGGCGGT | Template: pCoofy_mScarlet-I  agcggccgccTTTGTACAGTTCGTCCATACCGC |
